# Water Deficit Timing Affects Physiological Drought Response, Fruit Size, and Bitter Pit Development for ‘Honeycrisp’ Apple

**DOI:** 10.3390/plants9070874

**Published:** 2020-07-09

**Authors:** Michelle Reid, Lee Kalcsits

**Affiliations:** 1 Tree Fruit Research and Extension Center, Washington State University, Wenatchee, WA 98801, USA; michelle.reid@wsu.edu; 2 Department of Horticulture, Washington State University, Pullman, WA 99164, USA

**Keywords:** *Malus* x *domestica* Borkh., temperature, gas exchange, stem water potential, shoot growth

## Abstract

Irrigation is critical to maintain plant growth and productivity in many apple-producing regions. ‘Honeycrisp’ apple characteristically develops large fruit that are also susceptible to bitter pit. Limiting fruit size by restricting irrigation may represent an opportunity to control bitter pit in ‘Honeycrisp’. For three seasons, ‘Honeycrisp’ trees were subject to water limitations in 30-day increments and compared to a fully watered control. Water limitations were imposed from 16–45, 46–75, and 76–105 days after full bloom (DAFB). Soil moisture for the well-watered control was maintained at 80–90% of field capacity for the entire season. For two years, physiological measurements were made every 15 days from 30 to 105 DAFB. Fruit quality, bitter pit incidence, shoot length, and return bloom were also measured to assess impacts on growth and productivity. When water was limited, stomatal conductance and net gas exchange were lower compared to the well-watered control and stem water potential decreased by 30–50% throughout the growing season. Early season water limitations had a lower impact on plant response to abiotic stress compared to late-season limitations. Overall, water deficits during fruit expansion phases contributed to fewer large fruit and decreased overall bitter pit incidence with no negative effects on fruit quality.

## 1. Introduction

Irrigation is critical to maintain plant growth and productivity in many apple-producing regions. In the future, water limitations may drive the adoption of irrigation strategies that use less water but, at the same time, minimize reductions in fruit size. In the United States, ‘Honeycrisp’ apple production has rapidly expanded despite production challenges that can often include postharvest losses that exceed 50% [1]. ‘Honeycrisp’ fruit can be oversized, especially in younger trees. It also exhibits strong alternate bearing where fruit size can be even greater in a low-bearing year [2]. This oversized fruit can contribute to an increase in susceptibility to physiological disorders like bitter pit, a calcium-related disorder associated with mineral nutrient imbalances and low fruit calcium content. Bitter pit has been reported to correspond with fruit size because of the calcium dilution effect from fruit expansion [3]. These losses have an impact on the economic and environmental sustainability of apple production. Careful crop load management and frequent calcium sprays are current tools used to reduce the incidence of this disorder [4,5]. However, bitter pit continues to be a significant problem for ‘Honeycrisp’ in all growing regions. One alternative strategy to manage fruit size and, concomitantly, bitter pit incidence, may be the integration of irrigation management to reduce fruit size in addition to other best-used practices to control bitter pit.

Deficit irrigation is a practice by which less water is supplied to the plant than that of the expected evapotranspiration [6]. The inherent goal of deficit irrigation is to enhance production efficiencies that can be either achieved through reductions in applied irrigation or improved fruit quality. Previous research has tested several deficit irrigation periods that have included early season, late season, throughout the entire period of fruit development, or after harvest on several different tree fruit species. The response to water limitations can differ depending on the timing of deficit irrigation. Early season deficit irrigation in ‘Bartlett’ pear [7], ‘Delicious’ apple [8], and ‘Hosui’ Asian pear [9] reduced water use and vegetative growth while reporting minimal negative consequences to fruit size. Water stress early during fruit development at the time of fruit cell division can reduce cell numbers, resulting in smaller fruit [10]. For late season deficit irrigation, reduction in tree size was reported in ‘Housi’ Asian pear and ‘Braeburn’ apple [11,12]. However, the impact of deficit irrigation on apple trees with dwarfing rootstocks planted to high-density systems has not been as clearly desribed. 

Deciduous trees such as apple can respond differently to water stress depending on the developmental stage of the tree [13]. However, in general, when water is limited, decreases in stem and leaf potential stimulated by decreases in soil matric potential can promote a cascading biochemical response that contributes to changes in growth and plant development [14]. Even during short periods of water limitations, plant growth and development can slow or sometimes stop [15]. The response to water stress is a function of time and severity and both factors need to be considered when measuring plant response to water limitations. Furthermore, xylem cavitation during severe stress events can impair or delay recovery from drought even after irrigation has resumed [16].

Water limitations control water loss and photosynthesis by leaves. These responses are largely associated with changes in stomatal conductance. During scenarios where water is limited, decreases in photosynthesis are induced from stress response mechanisms including reduced CO_2_ conductance by stomatal closure [17]. However, the link between net gas exchange and leaf water potential is not direct. Photosynthesis has been reported to be less responsive to decreases in leaf water potential until the water potential passes a specific threshold [18] that may vary across species and cultivar. Once that threshold has been met, net gas exchange will decline as stomatal conductance decreases. During severe stress, abrupt declines in carbon assimilation can occur in conjunction with low leaf water potential because of irreversible damage to water transport mechanisms and photosynthetic machinery [18]. 

While plant responses to water limitations have generally been well described, the adoption of irrigation practices requires accurate measures of cultivar-specific responses to water limitations. Currently, soil moisture monitoring and evapotranspiration modelling are the primary methods by which irrigation decisions are currently made for apple [19,20]. However, one of the best indicators of physiological drought stress in tree fruit is plant water potential [21], which is often measured as mid-day stem water potential (Ψ_md_) [22]. While leaf water potential (Ψ_l_) can also be a good indicator of water stress, it can be environmentally plastic compared to stem water potential [23]. Environmental conditions have also been reported to affect measurements of stem water potential [24]. 

Identifying whole tree response to changes in plant water status may help make timely and accurate irrigation decisions to reduce water applications and possibly increase quality in ‘Honeycrisp’ apple. Because the physiological responses of ‘Honeycrisp’ to water limitations or other stress factors have not been adequately described, the impact of water limitations at different developmental periods must be better understood. Here, we sought to identify physiological responses for ‘Honeycrisp’ apple trees under early-, mid-, or late-season water limitations and to quantify the impact on fruit size and bitter pit incidence. We hypothesized that deficit irrigation that limits fruit size will reduce overall bitter pit incidence in Honeycrisp apple. This will provide direct information on the ability of ‘Honeycrisp’ apple to tolerate water limitations and how it affects fruit quality and disorder incidence. Providing identifiers of plant water status for ‘Honeycrisp’ will increase the adoption of irrigation practices, thus enhancing fruit quality for this high value cultivar.

## 2. Results

### 2.1. Soil Volumetric Water Content

**Figure 1:**
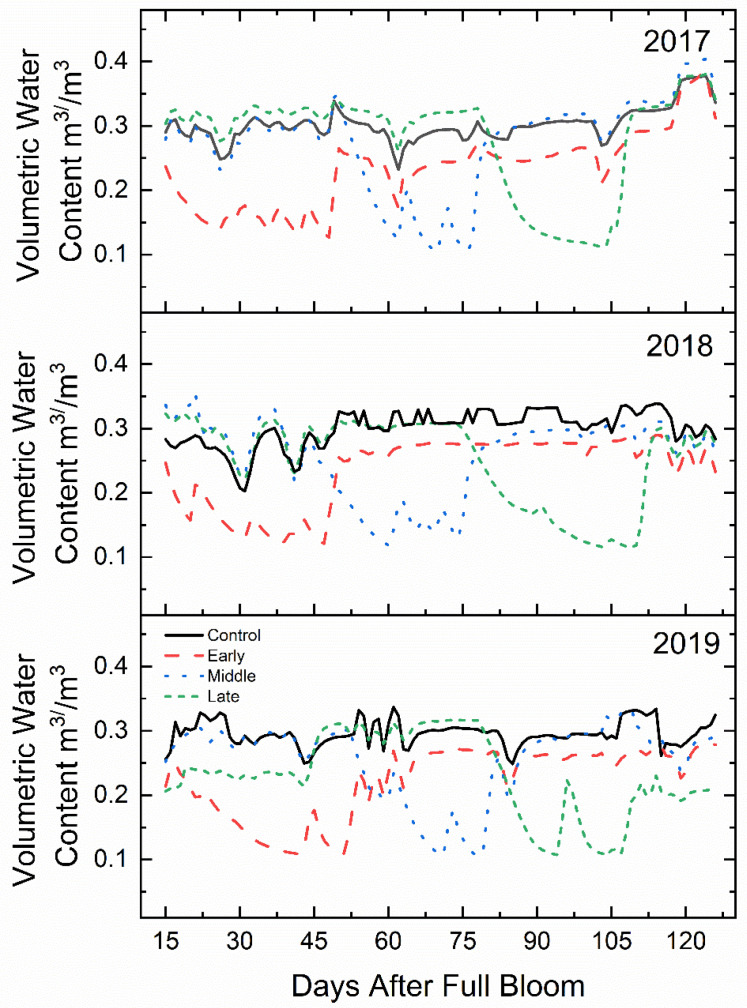
Mean soil volumetric water content (m^3^ m^−3^) (*n* = 3) of ‘Honeycrisp’ apple under deficit irrigation from full bloom to harvest in 2017 (bottom), 2018 (middle), 2019 (top). Four treatments were established including a well-watered control (line), early-season deficit (16–45 days after full bloom [DAFB]; dashes), mid-season deficit (46–75; dots), and late-season deficit (76–105 DAFB; small dashes).

The approximate field capacity of the soil in this experiment was determined to be 33% *v*/*v*. Soil volumetric water content was maintained between 80 and 90% of field capacity for non-limiting irrigation periods (Figure 1). During deficit treatments, the volumetric water content (m^3^ m^−3^) averaged between 35 and 45% of field capacity for all three years. The total water volume applied during the 30-day deficit periods were less than 10% of the normally watered control.

### 2.2. Shoot Growth, Crop Load, Return Bloom

**Figure 2:**
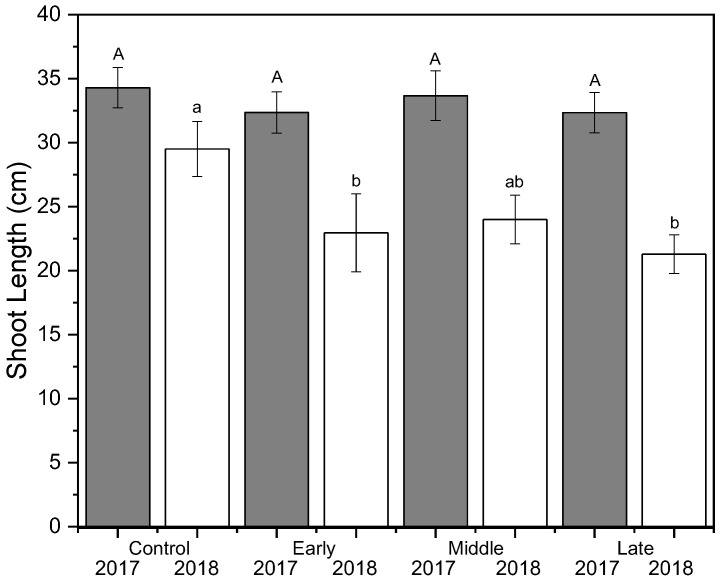
Mean shoot growth (cm) (±SE; *n* = 3) of ‘Honeycrisp’ apple under deficit irrigation in 2017 (gray) and 2018 (white). Four treatments were established including a well-watered control, early season deficit (16–45 days after full bloom [DAFB]), mid–season deficit (46–75 DAFB), and late season deficit (76–105 DAFB). Uppercase letters denote significance among treatments for 2017 and lowercase letters denote significance among treatments for 2018.

**Figure 3:**
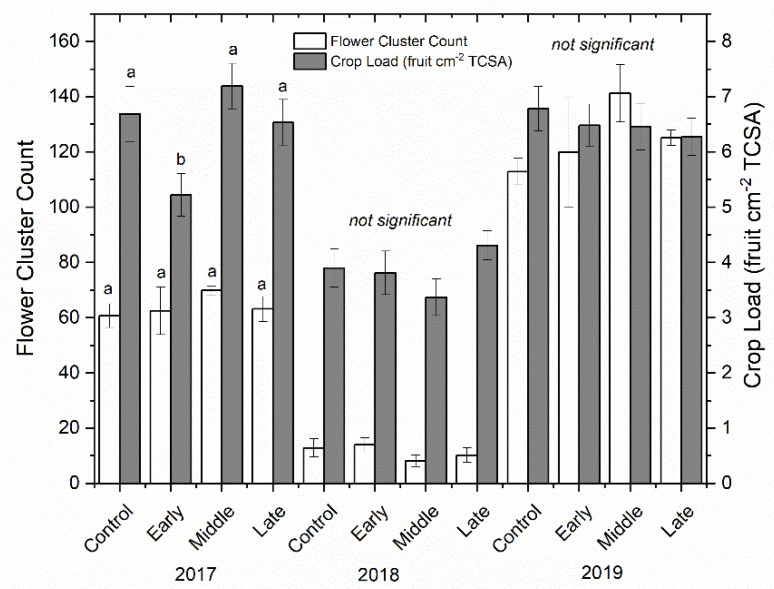
Mean bloom clusters per tree (cm) (±SE; *n* = 3) (left-axis; white bars) and crop load (right-axis; grey bars) for ‘Honeycrisp’ apple in 2017 (left), 2018 (middle), and 2019 (right). Four treatments were established including a well-watered control, early season deficit (16–45 days after full bloom [DAFB]), mid-season deficit (46–75 DAFB), and late season deficit (76–105 DAFB). Letters denote significance among treatments within each year determined using a Tukey’s HSD test (α = 0.05).

Shoot growth was significantly reduced by deficit irrigation in 2018 but not in 2017 (*p* < 0.001) (Figure 2). The well-watered control had significantly more shoot growth in 2018 compared to both early and late irrigation deficit treatments, but not compared to the mid-season deficit treatment. Return bloom was unaffected by irrigation treatments in both 2018 and 2019 (Figure 3). Mean bloom counts were 64, 11, and 125 bloom clusters per tree in 2017, 2018, and 2019, respectively. Crop load was standardized as much as possible using bloom thinners, post-bloom thinners, and hand thinning to the target crop load. Crop load averaged six fruit cm^−2^ trunk cross-sectional area (TCSA) in 2017 and 2019 and approximately four fruit cm^−2^ TCSA in 2018 (Figure 3). Crop load differences between years were much lower than differences in flower numbers. There were no differences in crop load among treatments except where trees that had early season deficit irrigation applied were overly thinned in the first year.

### 2.3. Net Gas Exchange

**Figure 4:**
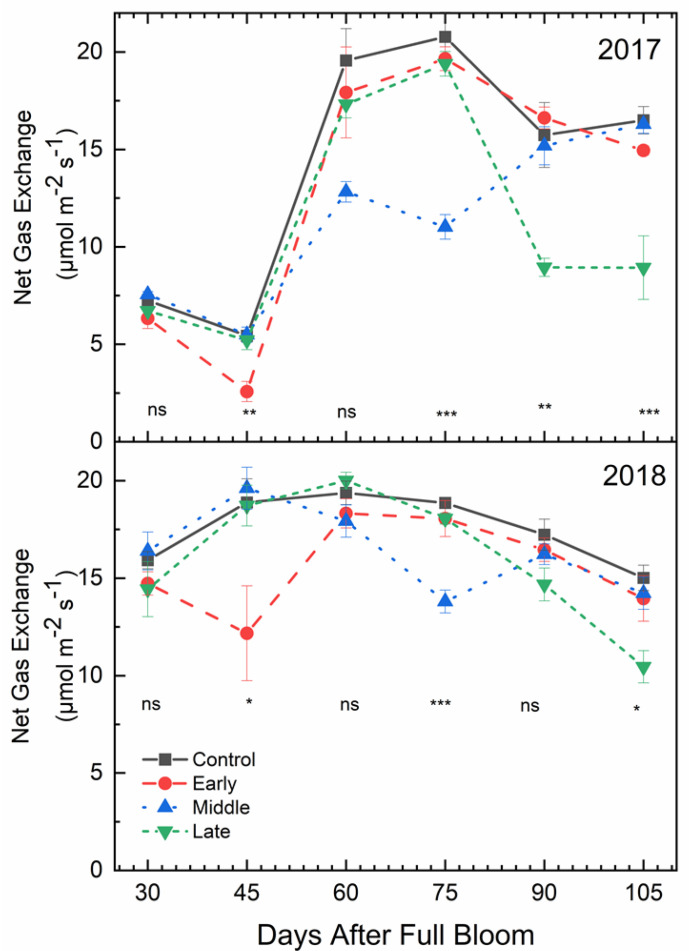
Mean mid-morning net gas exchange (μmol CO_2_ m^−2^ s^−1^) (±SE; *n* = 3) of ‘Honeycrisp’ apple under deficit irrigation in 2017 (bottom) and 2018 (top). Four treatments were established including a well-watered control (squares), early season deficit (16–45 days after full bloom [DAFB]; circles), mid-season deficit (46–75 DAFB; triangles), and late season deficit (76–105 DAFB; inverted triangles). Symbols denote significance among treatments at each measurement date (ns = not significant, * *p* ≤ 0.05, ** *p* ≤ 0.01, *** *p* ≤ 0.001).

Mid-morning net gas exchange rates (*P*n) were affected by deficit irrigation timing (*p* < 0.001) (Figure 4). In both 2017 and 2018, there were no significant differences in *P*n rates among treatments at 30 days after full bloom (DAFB), which was 15 days after the early season deficit treatment was initiated. However, for both years, *P*n rates were lower under early season deficit irrigation compared to the well-watered control at 45 DAFB, which was at the end of the early deficit treatment. In 2017, at 90 DAFB, 15 days after late-season deficit treatment was initiated, *P*n rates were lower under deficit irrigation compared to the well-watered control. However, there were no significant differences in *P*n rates at 60 and 90 DAFB in 2018. In both years, *P*n rates at were lower under deficit irrigation 75 and 105 DAFB, which were 30 days after the mid- and late-season irrigation treatments were initiated.

### 2.4. Stomatal Conductance

**Figure 5:**
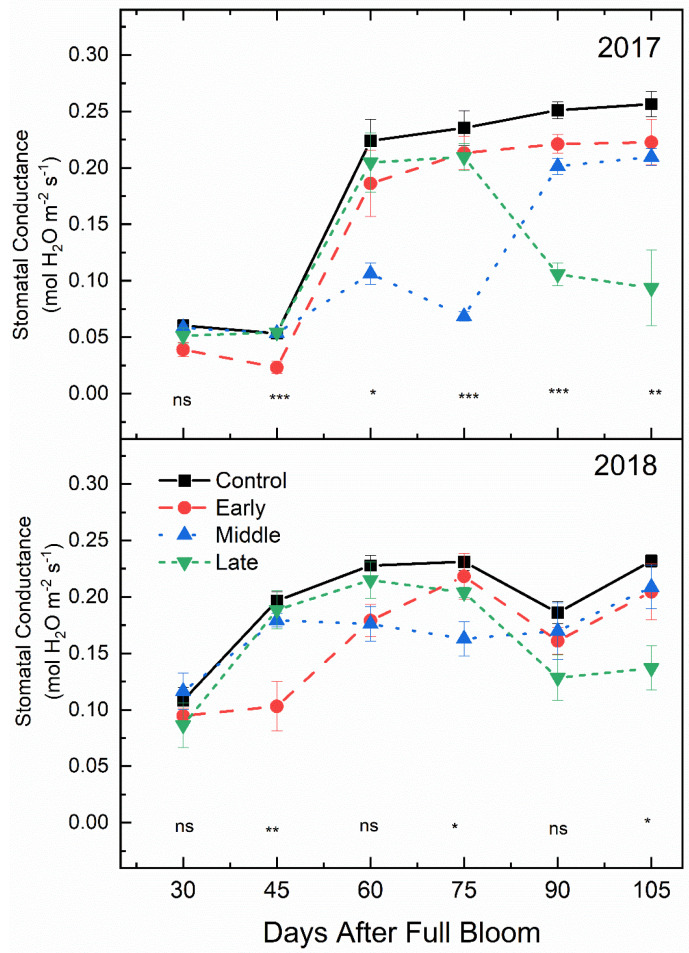
Mean mid-morning stomatal conductance (g_s_) (±SE; *n* = 3) of ‘Honeycrisp’ apple under deficit irrigation in 2017 (bottom) and 2018 (top). Four treatments were established including a well-watered control (squares), early season deficit (16–45 days after full bloom [DAFB]; circles), mid-season deficit (46–75 DAFB; triangles), and late season deficit (76–105 DAFB; inverted triangles). Symbols denote significance among treatments at each measurement date (ns = not significant, * *p* ≤ 0.05, ** *p* ≤ 0.01, *** *p* ≤ 0.001).

Mid-morning stomatal conductance was affected by deficit irrigation timing (*p* < 0.001) (Figure 5). For both years, stomatal conductance (g_s_) under early season deficit irrigation was almost half that of the well-watered control at 45 DAFB. At 60 DAFB, 15 days after the mid-season deficit treatment was established, g_s_ in the mid-season deficit treatment was significantly lower than the well-watered control for both years. g_s_ was also significantly lower at the end of the mid-season deficit treatment (75 DAFB) compared to the well-watered control. For both 2017 and 2018, g_s_ was significantly lower for leaves sampled from the late season deficit treatment compared to the fully watered control at 90 and 105 DAFB.

### 2.5. Mid-Day Stem Water Potential

**Figure 6:**
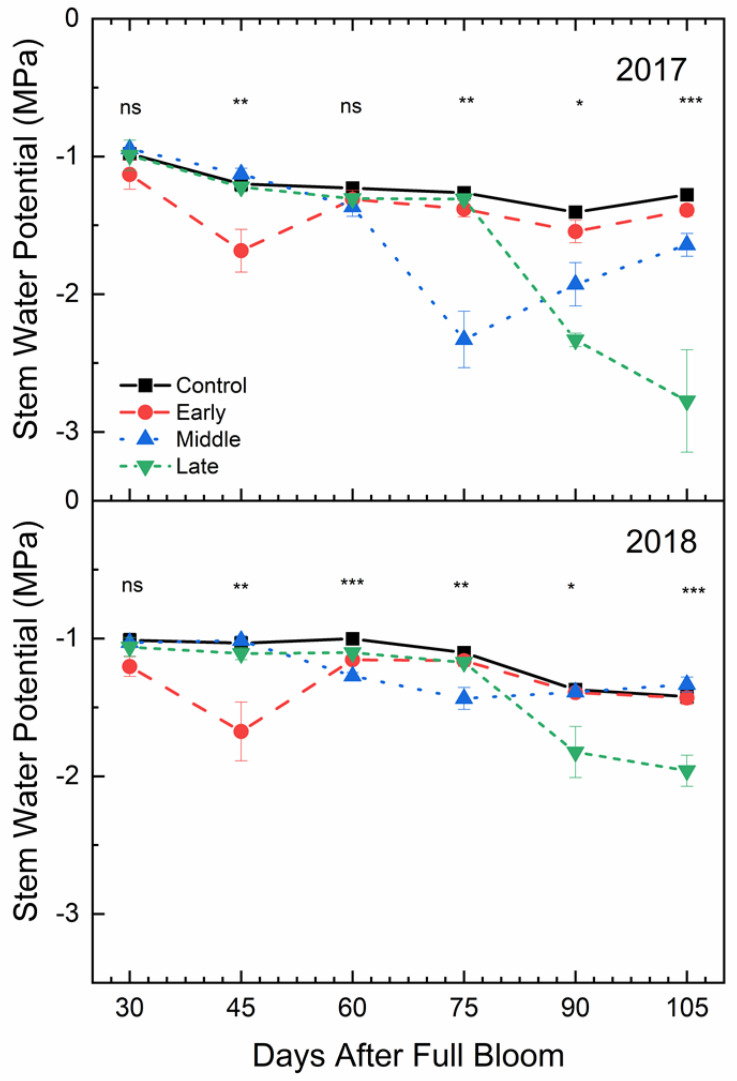
Mean mid-day stem water potential (±SE; *n* = 3) of ‘Honeycrisp’ apple under deficit irrigation in 2017 (bottom) and 2018 (top). Four treatments were established including a well-watered control (squares), early season deficit (16–45 days after full bloom [DAFB]; circles), mid-season deficit (46–75 DAFB; triangles), and late season deficit (76–105 DAFB; inverted triangles). Symbols denote significance among treatments at each measurement date (ns = not significant, * *p* ≤ 0.05, ** *p* ≤ 0.01, *** *p* ≤ 0.001).

Mid-day stem water potential was also affected by deficit irrigation treatments (*p* < 0.001) (Figure 6). In both years, Ψ_md_ in the well-watered control was not significantly greater than the early season deficit treatment at 30 DAFB, which was 15 days after the early season deficit treatment had begun. At the end of the early season deficit treatment, stem water potential in the well-watered control was significantly greater than the early season deficit. The Ψ_md_ for trees in the well-watered control was significantly greater than under mid-season deficit treatment at 75 DAFB, which was 30 days into the mid-season deficit treatment. Similar differences were seen in the late season deficit period. In the period following each deficit period, Ψ_md_ for deficit treatments were not significantly different from the control except in 2017 where the mid-season deficit treatment remained significantly lower at both 90 and 105 DAFB, even after full irrigation had been resumed.

### 2.6. Fruit Size, Quality, and Bitter Pit Development

**Figure 7:**
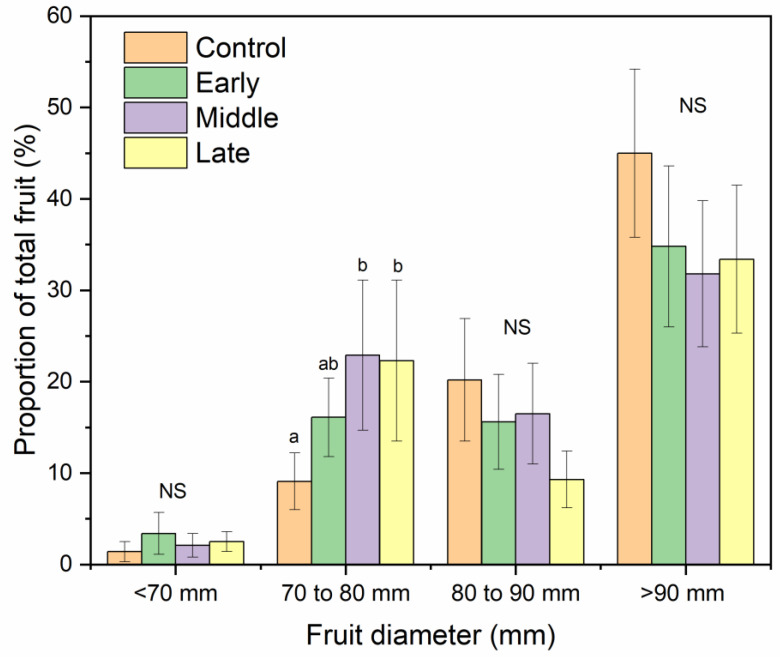
Percentage of Honeycrisp fruit belonging to each size category (<70 mm, 70–80 mm, 80–90 mm, and >90 mm diameter) for trees where early, middle, and late season water deficits were applied compared to a well-watered control from 2017 to 2019. NS = not significant among treatments within each size category. Letters denote significant difference among treatments determined using a Fisher’s LSD test (α = 0.05).

**Figure 8:**
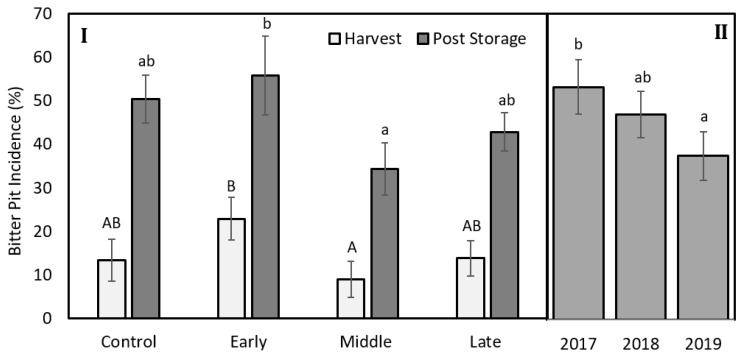
**I**. Percentage of total Honeycrisp fruit with bitter pit at harvest (light grey) and after three months of storage in regular atmosphere at 1 °C (dark grey) for trees where early, middle, and late summer water deficits were applied compared to a well-watered control. **II**. Bitter pit incidence after storage for fruit harvested in 2017, 2018, or 2019. Letters denote significant differences among treatments determined using a Fisher’s LSD test (α = 0.05).

**Figure 9:**
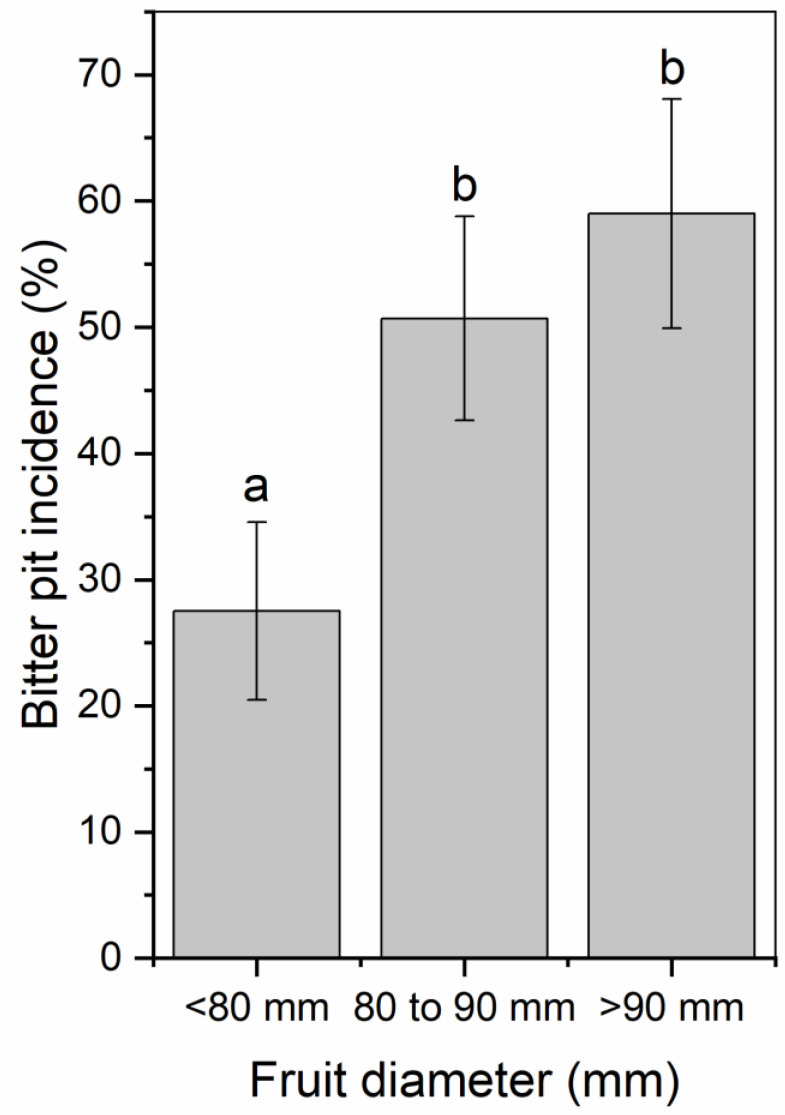
Percentage of total Honeycrisp fruit with bitter pit for fruit that was less than 80 mm, 80 to 90 mm, or greater than 90 mm in diameter after three months of storage in regular atmosphere at 1 °C. Letters denote significant differences between each size category determined using a Fisher’s LSD test (α = 0.05).

The mean fruit weight was 234, 299, and 280 g for 2017, 2018, and 2019, respectively (Table 1). Mean fruit weight was lower for fruit from trees exposed to late summer deficit irrigation in 2017. In 2017, fruit was firmer, had greater soluble solids content, and had greater red color development compared to the control and mid-season treatments (Table 1). Deficit irrigation during the middle and late summer shifted the size profile of harvested fruit. The percentage of fruit that were less than 80 mm in diameter was greater when water was limited either during the middle or late season (Figure 7). Bitter pit incidence was significantly greater in the early season deficit treatment compared to the middle and late season deficit treatments after storage (*p* < 0.05; Figure 8) but not different than trees that were fully watered all season. Since there were significantly higher proportions of fruit belonging to the 70 to 80 mm size category from the mid- and late-season deficit irrigation treatments, this accounted for the reductions in bitter pit incidence observed overall. Bitter pit incidence was the highest in 2017, the first year of production, and decreased in 2018 and then again in 2019 (Figure 8). As fruit size increased, bitter pit incidence also increased (Figure 9). For 2017–2019, fruit that was less than 80 mm in diameter had 27% of fruit with bitter pit after storage. However, fruit that was greater than 80 mm had more than 50% of fruit affected by bitter pit after storage. 

## 3. Discussion

**Figure 10:**
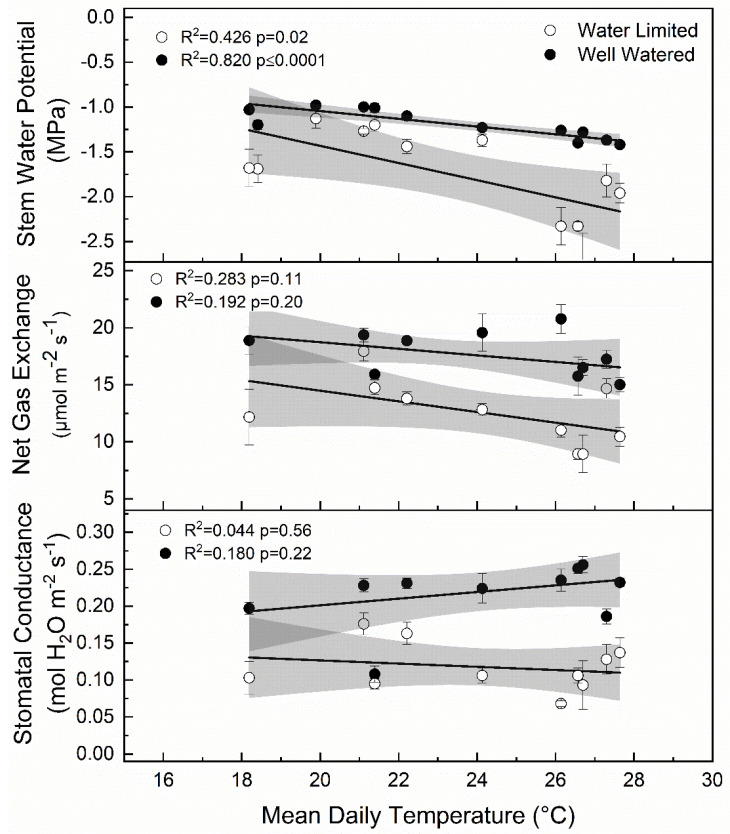
Linear relationship between mean daily temperature for 15 days prior to each measurement period and stomatal conductance, net gas exchange, and stem water potential for apple trees exposed to water limiting conditions (open circles) versus well-watered control (closed circles) in 2017 and 2018. Grey shaded sections represent the 95% confidence bands for the linear correlation shown by the black line.

Here, we report the response of ‘Honeycrisp’ apple to water limitations in a desert environment with minimal precipitation. Air temperature was a significant factor in tree response to deficit treatments (Figure 10). Specifically, stem water potential was negatively related to air temperature when trees were exposed to deficit irrigation. The controlled manipulation of plant water status via deficit irrigation interacted with air temperature to shape associated physiological responses in apple. In some cases, stomatal conductance, net gas exchange, and stem water potential were lower in the deficit treatments compared to the well-watered control 15 days into the deficit period, but all were significantly lower 30 days into each deficit period. After deficit irrigation periods, stress appeared to affect the rate of recovery. For deficit periods when stem water potential was lower, recovery was slower after water limitations indicated by significantly lower stem water potential even after normal irrigation had been resumed. 

### 3.1. Growing Season Environment Affects Plant Response to Water Limitations

In this study, significant differences in stem water potential between years were observed (*p* < 0.001). In 2017, the mean Ψ_md_ was −1.46 MPa, respectively whereas in 2018, Ψ_md_ was −1.28 MPa. Large differences in weather between years likely contributed to the strong physiological responses to water stress in 2017 that were not observed in 2018, when it was cooler during the mid-season period. In other studies, temperature, humidity, and wind speed have all been attributed to variable responses in stem water potential [22]. Plasticity in response to environment is important because all variables were held constant from year to year except the seasonal environmental conditions (Table 2). Immediately following bloom, at 0–15 DAFB, mean temperatures were 3.6 °C higher in 2018 and 2019 than 2017. More importantly, during the mid-season deficit at 46–75 DAFB, mean daily temperatures were 3.2 higher in 2017 than in 2018. The mean Ψ_md_ was −2.33 MPa in 2017 at 75 DAFB, versus −1.44 MPa in 2018. These differences in temperature appeared to affect stem water potential for trees that were under periods of deficit irrigation but not well-watered trees (Figure 10). Stem water potential for both well-watered and water limited trees showed a significant relationship with mean daily air temperatures recorded 15 days preceding each measurement (deficit r^2^ = −0.369 *p* = 0.02; well-watered r^2^ = −0.802 *p* < 0.0001). However, gas exchange measurements including net CO_2_ exchange and stomatal conductance showed no linear relationship with mean daily temperatures. Unsurprisingly, as air temperatures increased, there was a greater separation of measures of plant water status between well-watered and water limited trees.

### 3.2. Recovery from Drought Events Was Dependent on the Severity of Stress

As stress response increased, as indicated by low stem water potential, net gas exchange and stomatal conductance, the recovery rate appeared to decrease for mid-season water limitations. Specifically, in 2017, recovery from low stem water potential during the middle deficit irrigation period (46–75 DAFB) lasted for at least 30 days after the end of the deficit irrigation, possibly indicating some long-lasting effects on plant water status. This could possibly be due to cavitation events or that xylem anatomy may have been affected by water limitations [25]. Similar findings have been reported for forest species and modifications to xylem anatomy but there were species-specific responses that affected acclimation through these mechanisms [26]. Since these were short-term water limitations lasting only 30 days, we were not expecting large changes in xylem anatomy, and xylem cavitation may be more likely causing changes in recovery. We acknowledge that recovery was not directly measured in this study following late season water limitations. However, we avoided measuring water status immediately before and after harvest because of confounding effects of crop removal [27] or crop load [28,29] that could have affected measurements of plant water status. 

Here, ‘Honeycrisp’ appeared to have lower stem water potential values than other cultivars previously used in drought response studies. Previously, Ψ_md_ reference values of −1.3 MPa in ‘Mutsu’ and ‘Cox Orange’ apple were reported after 10 days of water-limited conditions [30]. In another study on ‘Gala’, irrigation was withheld until a −1.2 MPa threshold for Ψ_md_ was reached without any negative effects on yield or fruit quality [31]. In both years of this study, after 15 days for early, middle, and late water limitations, mid-day stem water potential was near the threshold reported in other studies. However, elevated vapor pressure deficits during the late season pushed even well-watered trees below the −1.2 MPa observed in other studies. After 30 days of water limitations, Ψ_md_ significantly surpassed the thresholds of −1.3 MPa and the trees did not show any visible symptoms of water stress such as wilting or leaf senescence [32]. This suggests that ‘Honeycrisp’ may demonstrate more stress tolerance than other apple cultivars that have been used for water relations research.

### 3.3. Stem Water Potential Is a Strong Indicator of Plant Water Status

Stem water potential was the most stable indicator of changes in plant water status. However, it was also the most responsive to temperature, which may have affected plant responses to changes in soil moisture. Although there was a general decrease in stem water potential as air temperatures increased (Figure 10), the response to deficit irrigation was consistent (Figure 6). Furthermore, the integration of environmental factors into the interpretation of stem water potential has implications on water management during different development periods or during rapidly changing environmental conditions during the growing season. In previous work using peach, the use of mid-day stem water potential along with predawn leaf water potential and cumulative transpiration rate allowed for the detection of water stressed trees at an earlier period than canopy transpiration or mid-day leaf water potential alone [33]. Although net gas exchange and stomatal conductance were also responsive to deficit irrigation in ‘Honeycrisp’ apple, these factors were more variable compared to stem water potential. Stomata are more sensitive to other environmental factors like vapor pressure deficit, which make stomatal conductance measurements not fully related to soil water content [34]. However, in this case, there was no significant relationship observed between air temperature and stomatal conductance (Figure 10). Among plant water status indicators, mid-day stem water potential has shown to have the best sensitivity to variability ratio [35]. Significant differences in mid-day stem water potential after 15 days of water limitations were seen (Figure 6), while stomatal conductance (Figure 5) and net gas exchange (Figure 4) generally did not show differences until 30 days of water limitations. Our findings agree with those reported by [36] whose work on *Prunus* sp. showed that when moderate deficits were applied, stem water potential was responsive, and recovery occurred quickly once water was reapplied.

### 3.4. Irrigation Deficits during Fruit Expansion Reduces the Number of Large Size Fruit That Are More Susceptible to Bitter Pit Development

Smaller fruit (<80 mm) had lower bitter pit incidence than larger fruit. There were greater proportions of fruit in smaller size categories when deficit irrigation was applied during either mid- or late-season periods. Fruit quality was relatively unaffected by irrigation treatments. This has also been reported from other regions for ‘Honeycrisp’ [2]. Only one year showed differences in fruit quality among treatments and that occurred in 2017 when late summer deficit irrigation had the smallest fruit and highest soluble solids content, red color development, and firmness. Similar patterns were observed in 2018 and 2019 but were not significantly different among treatments. These relationships have also been reported in other apple studies [8]. Since 2017 was the hottest year, particularly during the middle and late season, this may have enhanced the effects of deficit irrigation to have a greater effect on fruit quality than the other two years. Deficit irrigation treatments did not affect return bloom. However, bitter pit susceptibility also has other contributing factors that were controlled in this experiment including crop load [4]. In 2018, crop load was lower than in 2017 and 2019 (Figure 3) but overall bitter pit incidence was highest in 2017 and decreased in 2018 and then, again, in 2019. There were significant yearly biennial effects of bloom with low bloom counts in 2018 but did not appear to be related to overall bitter pit incidence. Honeycrisp production is known to be strongly biennial, especially early in production [2]. Furthermore, the location in the tree can have a significant impact on fruit size and bitter pit incidence [37]. Although decreases in fruit diameter is a negative consequence of deficit irrigation for many apple cultivars, for ‘Honeycrisp’, fruit size can be excessively large in an irrigated system and excess size does not produce a premium return for producers. Therefore, bitter pit can be reduced through reducing fruit size by limiting water deliver during the critical fruit expansion phases.

## 4. Materials and Methods

### 4.1. Experimental Site and Tree Management

The experiment was conducted from 2017 to 2019 at the WSU Sunrise Research Orchard located in Rock Island, WA using ‘Honeycrisp’ trees grafted on M9-T337 rootstock planted in 2015 and trained to a tall spindle system. The orchard was located in a semi-arid environment with a mean annual precipitation of 231 mm. The planting density was 0.91 × 3.66 m (2989 trees ha^−1^) and oriented north to south. The soil was an alluvial shallow sandy loam soil. The mean daily maximum temperatures for 0–15, 16–45, 46–75, 76–105, and 106–125 DAFB in 2017, 2018, and 2019 are shown in Table 2.

Using a completely randomized design, irrigation regimes withheld irrigation either early, middle, or late in the season and were compared to a fully watered control with three replications for each treatment. Bloom occurred on 3 May 2017, 27 April 2018, and 26 April 2019. Lime-sulfur bloom thinning treatments were applied to limit fruit set at bloom. Trees were hand thinned in the middle of June for all three years. The orchard was harvested on 6 September 2017, 31 August 2018, and 6 September 2019. Trees were not sprayed with calcium in 2017. In 2018 and 2019, calcium was applied through four applications of calcium chloride at a rate of 4.47 kg ha^−1^.

### 4.2. Irrigation Treatments

The irrigation system was controlled with a variable speed pump drive and an electrovalve controller that enabled precise timing of irrigation applications. Using exclusion valves and by-pass lines, the entire block was randomized into 20-tree replications. Irrigation was applied using emitters spaced 0.3 m apart with a rate of 1.6 L ha^−1^ and supplemented with microsprinkler irrigation during treatment transition periods to maintain the grass between rows. Water was applied to the well-watered control in amounts that exceeded water demand with a daily schedule of four 30-min applications each day. The early season irrigation deficit was from 16 to 45 DAFB (during cell division), mid-season irrigation deficit was from 46 to 75 DAFB (early fruit expansion) and late season irrigation deficit was from 76 to 105 DAFB (late fruit expansion). All treatments followed the well-watered irrigation schedule of the control treatment before and after each deficit treatment period. Soil volumetric water content and soil temperature were measured with an ECH_2_O 5TM soil moisture and temperature probes (Decagon Devices, Pullman, WA, USA) placed 20 cm below the soil surface in each replicate in the weed-free herbicide strip directly between trees. Each probe was interfaced with an EM50G cellular data logger (Decagon Devices, Pullman, WA, USA) and data was logged every 30 min from full bloom to harvest. 

Volumetric soil moisture content was maintained at approximately 30–40% of field capacity when deficit irrigation was applied and approximately 80–90% of field capacity for the well-watered control and when deficit was not being applied for other treatments. Field capacity was determined using a soil sample that was watered to saturation, allowed to gravimetrically drain for two hours then weighed. The sample was then oven dried and then weighed again. From these two weights, field capacity was determined to be 33% *v*/*v*. During deficit periods, when the volumetric water content fell below 30% of field capacity, water was applied in small amounts to ensure that soil volumetric water content did not exceed 50% of field capacity. During these small irrigation pulses, deficit trees received four applications of 30 min each during a four-hour period.

### 4.3. Tree Selection, Growth Measurements and Return Bloom

Three sample trees from each replicate were selected for uniformity by measuring trunk diameter and assessing visual uniformity of tree growth. Caliper measurements were taken 15 cm above the graft union on all trees to determine TCSA. From this information, the sample trees for each replicate were selected to be within 10% of the mean TCSA of all 240 trees. Bloom clusters were counted to ensure uniformity of crop load in each year for sample trees. Shoot growth (cm) of 10 lateral shoots per sample tree were measured after harvest in both 2017 and 2018 to evaluate differences in shoot growth length between treatments. Although bloom in 2017 was not affected by deficit treatments, bloom was counted in 2017 to identify target crop loads and thinning targets. Return bloom was counted in April 2018 and 2019 to determine irrigation effect on return bloom. The TCSA was measured again in 2018, 15 cm above grafting union, to help calculate crop load and thinning targets.

### 4.4. Ecophysiological Measurements

Whole plant ecophysiology measurements were made every 15 ± 2 days beginning 30 DAFB until 105 DAFB on sample trees including net gas exchange, stomatal conductance, and mid-day stem water potential to evaluate plant response to deficit irrigation at different times of the growing season. Net gas exchange and stomatal conductance were measured on cloudless days between 10:00 a.m. and 12:00 p.m. using an LI-6400XT infrared gas analyzer fitted with a fluorescence head (Li-COR, Lincoln, NE, USA). Reference CO_2_ concentration was set at 400 ppm, leaf temperature at 25 °C, and photosynthetic photon flux density at 1500 μmol m^−2^ s^−1^. Measurements were made on two separate sun-exposed healthy leaves per tree between fifth and seventh leaf from the apical meristem. Once placed in the gas exchange chamber, each leaf was allowed to equilibrate until reference and sample values stabilized. 

Plant water status was measured as mid-day stem water potential (Ψ_md_) using a PMS 615D plant water potential console (Edaphic Scientific, Port Macquarie, NSW, Australia). Two healthy, mature leaves from inside the canopy nearest to the base of the tree were used for measurement of Ψ_md_. Each leaf was placed in a silver foil bag while still connected to the tree for at least one hour before measurement, to allow the leaf and xylem water potential to equalize before measurements were made. Ψ_md_ was measured at solar noon immediately after removing the leaves from each tree. Leaves were kept in silver foil bags and pressurized in a chamber until a droplet of sap formed on the cut leaf petiole. Stem water potential was taken as the inverse of the pressure to form a sap droplet on the leaf petiole [29,36].

### 4.5. Fruit Quality, Size, and Bitter Pit Assessment

At harvest, all fruit was picked from the sample trees on 6 September 2017, 31 August 2018, and 6 September 2019. The total amount of fruit from each tree was counted and weighed in the field. Fruit diameter was measured using a caliper and then was categorized into either <70, 70–80 mm, 80–90 mm, or >90 mm diameter classes. Per replicate, 30 fruit were randomly selected and used for fruit quality assessments at harvest and 30 fruit was placed in regular atmosphere (RA) storage for three months and then evaluated for bitter pit incidence. At harvest, fruit was weighed and then evaluated for color development. Color classification (1–4) was based on the coverage of red color on the fruit surface, 1 being 0–25% of red color covering fruit, 2 being 26–50%, 3 being 51–75%, and 4 being 76–100% and was also rated for bitter pit incidence. Fruit firmness was measured by removing the peel on opposite sides of the equatorial region of the fruit and then using a fruit texture analyzer (Güss Manufacturing Ltd, Strand, South Africa) with a 10 mm probe. A small section of fruit (1 cm^3^) was taken from the remaining portion of the top piece of fruit and was pressed in a garlic press onto the measurement surface of an Atago (3810) handheld refractometer (Bellevue, WA, USA) to estimate soluble solids content. 

### 4.6. Statistical Analysis

Data were analyzed as a completely randomized design using one-way analysis of variance (ANOVA) in OriginPro 9.1 software (Originlab Corporation, Northampton, MA, USA) with treatment as the main factor for net gas exchange, stomatal conductance, and mid-day stem water potential within each day of measurement (*n* = 3). End of season shoot growth data were analyzed as a one-way ANOVA with treatment as the main factor. Mean separation was performed for treatment differences within sampling days using Fisher’s least significant difference test (α = 0.05).

## 5. Conclusions

Water limitations during cell expansion reduced fruit size. Smaller fruit had lower bitter pit incidence compared to larger fruit. Deficit timing did not negatively affect fruit quality at harvest or return bloom for the following season. Stem water potential appeared to integrate environmental pressures with soil moisture better than other measures such as gas exchange or stomatal conductance. Deficit irrigation in semi-arid environments requires efficient indicators of plant water status to limit risk and increase the precision of water applications. With environmental conditions that change annually, understanding the dynamic physiological responses of ‘Honeycrisp’ apple to water-limited conditions provided a better understanding of its response to water limitations and its interaction with air temperature. Regulated deficit irrigation can be a tool used to limit excessive fruit size and, subsequently, can reduce bitter pit incidence in ‘Honeycrisp’ apple.

## Figures and Tables

**Table 1:** Fruit quality after three months of storage in regular atmosphere at 1 °C for trees where early, middle, and late summer water deficits were applied compared to a well-watered control. Different letters denote significant differences within each year among treatments determined using a Fisher’s LSD test (α = 0.05).

Treatment	Weight (g)	Color Class (1–4)	Firmness (lb)	Soluble Solids Content (Brix)
**2017**				
**Control**	255 a	2.6 b	17.1 a	13.8 a
**Early**	247 ab	2.7 ab	17.4 a	15.3 c
**Middle**	226 b	2.3 b	18.1 a	14.5 b
**Late**	209 c	2.9 a	19.6 b	15.6 c
**2018**				
**Control**	326 a	2.7 a	16.0 a	14.9 a
**Early**	269 a	2.9 a	16.4 ab	15.7 a
**Middle**	299 a	2.8 a	17.4 b	15.4 a
**Late**	305 a	3.0 a	16.3 ab	15.2 a
**2019**				
**Control**	290 a	3.6 a	14.1 a	13.5 a
**Early**	276 a	3.2 a	14.0 a	13.6 a
**Middle**	278 a	3.6 a	14.1 a	13.2 a
**Late**	274 a	3.8 a	14.1 a	14.1 a

**Table 2:** Average weather conditions during specific growing days including minimum, average, and maximum temperature, relative humidity (RH), wind speed, total precipitation, total solar radiation, and vapor pressure deficit from full bloom to harvest at Sunrise Research Orchard in Rock Island, WA.

Year	DAFB	Min. Temp (°C)	Mean Temp (°C)	Max. Temp (°C)	RH (%)	Wind Speed (m s^−1^)	Total Precip. (mm)	Total Solar Radiation (MJ m^−2^)	Vapor Pressure Deficit (kPa)
**2017**	0–15	7.4	13.9	20.5	56.1	2.3	20.1	331	0.70
	16–45	12.1	19.3	26.5	44.7	2.6	7.6	703	1.24
	46–75	17.3	24.9	33.0	33.1	3.1	0.0	820	2.11
	76–105	18.9	26.6	34.9	32.5	2.7	2.0	699	2.35
	106–125	16.2	23.9	32.8	39.3	2.2	0.0	379	1.80
**2018**	0–15	11.5	17.6	24.1	46.8	3.1	7.4	334	1.07
	16–45	13.3	19.8	27.0	40.9	3.1	29.5	720	1.37
	46–75	14.3	21.7	28.8	39.8	3.1	7.1	758	1.56
	76–105	19.3	27.5	36.2	30.4	2.7	0.0	749	2.56
	106–125	15.2	22.5	30.4	40.8	2.4	0.3	335	1.61
**2019**	0–15	9.5	17.6	24.8	34.8	2.6	0.0	356	1.31
	16–45	13.3	19.6	26.4	45.1	2.7	10.4	631	1.25
	46–75	15.8	21.8	28.8	40.1	3.2	13.0	719	1.57
	76–105	17.1	24.4	32.2	38.5	2.7	19.0	692	1.88
	106–125	17.1	24.0	31.8	40.3	2.62	0	398	1.78
